# What drives waste sorting? A capability, opportunity, motivation, and behavior model analysis with hybrid modeling

**DOI:** 10.3389/fpsyg.2025.1625538

**Published:** 2025-09-15

**Authors:** Beijia Zhang

**Affiliations:** School of Management, Anhui University, Hefei, Anhui, China

**Keywords:** waste sorting behavior, capability, opportunity, motivation and behavior model, structural equation modeling, system dynamics, China

## Abstract

To motivate urban residents to actively participate in waste sorting, this study aims to clarify the behavioral mechanisms driving residents' waste sorting behavior. Based on the Capability-Opportunity-Motivation-Behavior (COM-B) model, a comprehensive approach combining Structural Equation Modeling (SEM) and System Dynamics (SD) was employed to analyze the sample of Shanghai residents, examining the static and dynamic relationships between various factors and waste sorting behaviors. Results show that capability (knowledge), opportunity (infrastructure, subjective norms), and motivation (habit and intention) are positively correlated with sorting behavior, with the opportunity also has significant indirect effects on behavior via motivation. Notably, opportunity factors demonstrated stronger effect than capability and motivation. And when levels of capability, opportunity, and motivation increase over time, waste sorting behavior exhibits a nonlinear growth trend, accelerating from slow to fast. Therefore, in the cycle management of waste sorting, emphasis should be placed on later-stage efforts, prioritizing interventions that enhance opportunity factors to promote sustainable sorting behaviors. These findings offer theoretical and practical guidance for urban waste sorting management efforts.

## 1 Introduction

China introduced the *Household Waste Sorting System Implementation Plan* in 2017, which demonstrated a significant increase in waste sorting efforts (Luo et al., 2022). The waste sorting behavior of urban residents involves categorizing household waste according to regulations and placing each type in designated areas for proper treatment (Areeprasert et al., 2018). As the first segment in waste disposal, its effectiveness directly impacts the efficiency of subsequent collection, transportation, and treatment, and is essential for achieving waste reduction, harmlessness, and resource recovery (Peng et al., 2021).

To encourage the deployment of waste sorting among urban residents and increase their participation rate in sorting, it is critical to determine the fundamental elements relevant to waste-sorting behavior (Zhang et al., 2021). While some factors associated with residents' waste sorting have been identified, further research is needed to explore whether a new theoretical framework can better explain the behavior determinants, how these factors interact, and how their relationships evolve over time.

This study is based on the Capability, Opportunity, Motivation, and Behavior (COM-B) model, which develops a model of factors linked to urban residents' waste-sorting behavior, conducts empirical and simulation analyses to reveal key contributing factors, and explores the behavior's dynamic change patterns.

## 2 Literature review

### 2.1 Factors associated with waste sorting

To promote waste sorting among urban residents, researchers have long studied contributing factors and their relationships to identify appropriate interventions. Research on determinants of residents' waste-sorting behavior is mostly based on three perspectives: demographic, psychological, and contextual.

The most typical demographic factors used in studies on waste-sorting behavior are age, gender, education, and income. However, owing to the unpredictability of the statistical samples, the association between demographic factors and resident waste sorting behavior could not be reliably determined. For example, Vining and Ebreo (1990) suggested that older people are more likely to participate in categorical recycling, although Gamba and Oskamp (1994) found a minor negative correlation, and Oskamp et al. (1991) observed no relationship. Domina and Koch (2002) explained this discrepancy in generational differences.

Over the last few decades, social psychologists have developed several theoretical frameworks to analyze waste sorting behaviors and their antecedents. Previous studies highlighted the importance of attitudes, moral norms, and knowledge as psychological factors. According to the Theory of Planned Behavior (TPB), attitude is defined as a person's level of approval or disapproval of a specific behavior (Ajzen, 1991). Ari and Yilmaz (2016) found that attitude explains 60% of sorting behavior, showing that the residents' individual views about waste sorting had effect on their actual conduct, the conclusion shared by previous studies (Miafodzyeva and Brandt, 2013; Knickmeyer, 2020). According to the Value-Belief-Norm (VBN) paradigm, waste sorting is an environmental behavior motivated by moral norms (Xu et al., 2016). Loan et al. (2017) found empirical support for moral norms as a determinant of waste-sorting behavior. Wang et al. (2020) also stated in their study that residents with limited knowledge of waste sorting categories were less motivated to participate in waste sorting.

Contextual factors include environmental elements, such as policy, subjective norms, and convenience. Appropriate policy incentives, such as information disclosure, education, publicity, and role modeling, can help raise waste sorting levels (Schultz et al., 1995; Grazhdani, 2016). Social influence from family, neighbors, and friends encourages waste sorting and supports follow-up actions (Patrícia et al., 2004). As waste sorting by urban residents is a family behavior, internal family members may exhibit stronger behavioral linkages (Ting and Cheng, 2017). Xu et al. (2017) discovered that even minor modifications to enhance convenience can have a significant impact on behavior. Convenient, clean, and user-friendly waste collection stations promote waste classification by residents (Knickmeyer, 2020).

By reviewing studies on the factors associated with waste sorting behavior, it was found that many factors stem from multiple theories, which often identify associations but lack guidance for designing effective interventions from a theoretical standpoint. This highlights the need for a theoretical framework that integrates multiple factors and links them to interventions as the basis of analysis.

### 2.2 COM-B model

The COM-B model offers a comprehensive framework for integrating multiple theories and supporting behavioral intervention designs. It serves as the core behavioral layer of the Behavior Change Wheel Theory and connects to interventions in the peripheral intervention layer, to offer a concise, comprehensive, and logically coherent model for designing new behavioral interventions and describing existing ones (Olander et al., 2016).

The COM-B model states that behavior is the outcome of the interplay between capability, opportunity, and motivation, with capability and opportunity interacting with motivation (Michie et al., 2014; Ellis et al., 2019). Initially, the COM-B model was widely utilized in the medical industry, such as to investigate factors relevant to patient self-care behavior (Zou et al., 2017). Later, it was gradually introduced into other fields such as energy-saving behavior (Azizi et al., 2019), food safety (Thaivalappil et al., 2018), and invasive species management (Mcleod et al., 2015).

In recent years, several scholars have introduced the COM-B theory into the field of waste recycling behavior analysis. Non-quantitative questionnaires and interviews were commonly used in these research findings. The collected textual content was coded using the COM-B framework, which identified influential elements across three dimensions: capability, opportunity, and motivation. For example, Allison et al. (2021) and Cook et al. (2023) identified determinants of disposable paper cup use and hospital food waste recycling behavior. Fernandez-Alvarez et al. (2025) conducted quantitative research by developing a Spanish-language version of a COM-B framework-based factor quantification questionnaire for home waste recycling. The questionnaire passed reliability and validity testing, indicating acceptable psychometric qualities. Allison et al. (2022) conducted regression analysis to quantify associations between capability, opportunity, and motivation factors on household food waste recycling behavior.

Existing studies exploring associated factors with waste sorting behavior based on the COM-B framework have mostly focused on qualitative analysis, primarily identifying and describing potential associated factors. Even studies employing quantitative methods have largely been limited to investigating direct, static associations between factors and behavioral outcomes. This analytical perspective has two limitations: first, it fails to highlight the potential interacting processes among the numerous COM-B elements; second, it is unable to depict the dynamic evolution of waste sorting behavior and its associated factors over time. In real-world circumstances, changes in residents' behavior are dynamic and evolutionary, driven by a variety of factors. It is critical to investigate the mechanisms by which these factors interact and relate to behavioral evolution throughout time.

To address these limitations, this study will integrate the Structural Equation Modeling (SEM) and System Dynamics (SD) methods based on the COM-B model to analyze the static structural relationships among contributing factors, and then simulate the dynamic evolution of waste sorting behavior under the effect of multiple factors, thereby characterizing behavioral mechanisms from both static structural and dynamic evolutionary dimensions.

## 3 Theoretical model and hypotheses

**Figure 1:**
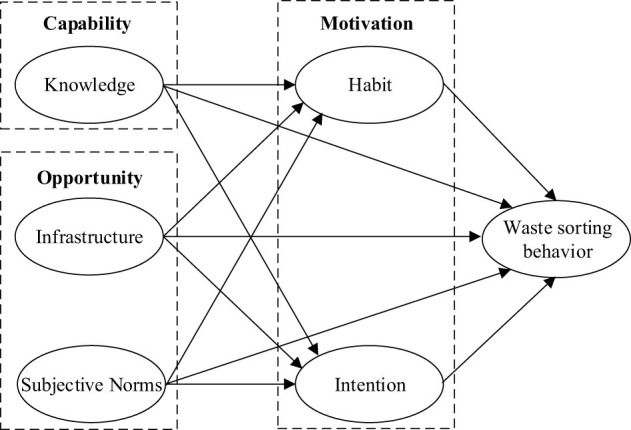
The conceptual model for waste sorting behavior.

This study developed an integrated research model based on the COM-B theoretical model to investigate the relationship between capability, opportunity, and motivation, as well as their associations with residents' waste-sorting behavior. Figure 1 illustrates the research model as follows:

### 3.1 Capability and behavior

Capability is described as an individual's psychological and physical ability to engage in the action at hand, including the possession of essential knowledge and skills (Michie et al., 2011). Knowledge demonstrates a significant statistical association with waste-sorting behavior. According to Chai and Baudelaire (2015), knowledge may be divided into two categories: “know what” and "know how.” The former refers to understanding the significance of trash sorting, whereas the latter refers to knowing the process itself. In some developed countries such as Australia and the United Kingdom, waste is classified as 60 percent. In comparison, developing nations, such as Iran and Turkey, have waste classification rates of less than 10 percent (Pakpour et al., 2014; Fan et al., 2019). This is because in many developing nations limited resident knowledge about waste limits it's sorting (Zand et al., 2020). Therefore, this study proposes the following hypothesis:

H1: Knowledge is positively related to waste sorting behavior.

### 3.2 Opportunity and behavior

Opportunity is defined as all the external variables that enable or urge an activity. (Michie et al., 2011). Here, opportunity consists of the infrastructure provided by the physical environment and the subjective norms provided by the sociocultural environment. Indeed, the lack of easily accessible infrastructure could be one of the most significant hurdles to individual waste sorting efforts. The number, distance, and appearance of sorting infrastructures play crucial roles in determining how easily residents can utilize them (Thomas and Sharp, 2013; Varotto and Spagnolli, 2017). Residents may take more sorting actions if proper infrastructure is easily accessible.

Subjective norms are the degree to which people perceive societal expectations relevant to their behavior (Sarkis, 2017). If the people around them are trustworthy or have leadership qualities, their opinions may correlate with behavioral choices (Shin and Hancer, 2016). For example, Park and Ha (2014) found that residents were motivated when they observed their neighbors or friends sorting and recycling waste. As a result, when residents' family members, acquaintances, and neighbors sort waste, it has a modeling effect on them, increasing the likelihood that residents will act in a similar manner. In this context, the study proposes two more hypotheses:

H2: Infrastructure is positively related to waste sorting behavior.
H3: Subjective norms is positively related to waste sorting behavior.

### 3.3 Motivation and behavior

Motivation refers to all brain processes that motivate and direct behavior, including habitual and emotional responses, as well as analytical decision-making processes (Michie et al., 2011). The change from the old waste disposal strategy of employing a single trash can to “automatic” waste sorting into various containers (Timlett and Williams, 2009) necessitates significant intrinsic incentives. The motivation for waste sorting can be divided into two types: habit that arises spontaneously and behavioral intention that emerges after considerable thought. Waste sorting, a daily practice conducted under comparable conditions, has the potential to become a habitual practice. Once waste sorting becomes a spontaneous habit, it becomes simple to develop repetitive behaviors; hence, habit is an important variable that supports long-term behaviors (Broers et al., 2021).

Behavioral intention is a precursor to actual conduct and is related to an individual's subjective judgment that particular behaviors are likely to occur in the future (Fishbein and Ajzen, 1977). Waste sorting intention has thus a significant predictive effect on behavior (Zhang et al., 2021; Lou et al., 2024). When the prerequisites for actual conduct are met, behavioral intention becomes actual behavior. Based on the above literature, this study proposes the following hypothesis:

H4: Habit is positively related to waste sorting behavior.
H5: Intention is positively related to waste sorting behavior.

### 3.4 Capability, opportunity, and motivation

According to the COM-B model, motivation mediates the relationship between capability, opportunity, and behavior. Prior studies have also provided evidence confirming the link between knowledge, subjective norms, habit, and intention (Li et al., 2017; Zhang et al., 2021). More hypotheses thus emerge from the suggestions by prior studies:

H6: Knowledge is positively related to habit.
H7: Knowledge is positively related to intention.
H8: Infrastructure is positively related to habit.
H9: Infrastructure is positively related to intention.
H10: Subjective norms is positively related to habit.
H11: Subjective norms is positively related to intention.

## 4 Methodology

**Figure 2:**
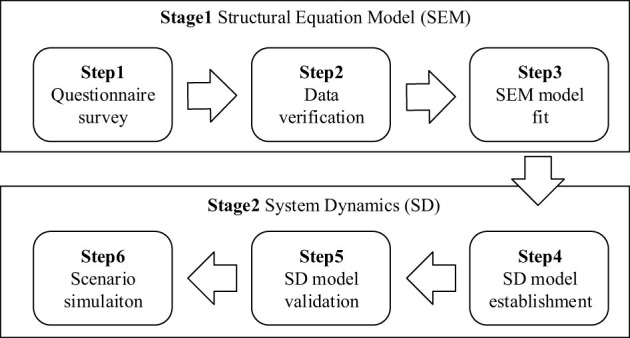
Two-stage research process.

This study used SEM and SD methodologies to investigate the processes associated with municipal waste sorting behavior. Although SEM can be used to verify theoretical relationships, the model only produces static associations between variables and cannot dynamically track how behavioral patterns co-vary with parametric modifications. SD can be used to investigate dynamic interconnections between variables, but the method requires substantial efforts to quantify the variables and equations using expert knowledge (Li et al., 2008). Using SEM model parameters to generate the SD model can help eliminate the errors and biases associated with expert judgment. Figure 2 illustrates each step in the two-stage research process.

### 4.1 Structural equation model (SEM)

#### 4.1.1 Step 1: questionnaire survey

The questionnaire consisted of three parts. The first explained key terms like household waste, waste sorting, and related infrastructure to ensure participants understood the context. The second measured six latent variables—knowledge (KI), infrastructure (IF), subjective norms (SN), habit (HA), intention (IN), and waste sorting behavior (WSB)—using a five-point Likert scale. The third section collected demographic information, including gender, age, education, and income. Details of the items are provided in Appendix A.

From January 4 to 26, 2025, data were collected via the Chinese Professional Survey website (https://www.wjx.cn/). Since Shanghai was among the first cities in China to mandate waste sorting and serves as a national model, so its residents were surveyed. Participants were assured of anonymity and that data would be analyzed in aggregate. A total of 589 responses were received, with 35 excluded for non-compliance, resulting in 554 valid questionnaires and a 94.1% valid response rate—over 10 times the number of measured variables (Fornell and Larcker, 1981).

#### 4.1.2 Step 2: data verification

The implementation of SEM requires scale data that satisfy the reliability and validity requirements. To conduct the reliability test, SPSS23.0 software was used to calculate Cronbach's α for each latent variable. If Cronbach's α > 0.7, the scale is highly reliable (Kline, 1998).

To verify construct validity, a confirmatory factor analysis (CFA) was performed using Mplus 8.3 software. Structural validity can be assessed using three criteria: (1) each measurable variable's factor loading should surpass 0.6; (2) each latent variable's composite reliability (CR) should exceed 0.7; and (3) the average variance extracted (AVE) for each latent variable should be more than 0.5 (Fornell and Larcker, 1981).

Finally, the square root of the AVE for each latent variable was compared to its correlation coefficient with the other latent variables. If the square root of the AVE exceeded the correlation coefficient, the scale showed strong discriminant validity (Gefen et al., 2000).

#### 4.1.3 Step 3: SEM model fit

Following the validation of the scale data, the model was fitted using Mplus 8.3 software, and the goodness of fit was evaluated to confirm that the model was statistically tractable and capable of producing stable and reasonable outcomes (Wu et al., 2015). The model was evaluated using a combination of the chi-square/degrees of freedom (CMIN/DF), root mean square error of approximation (RMSEA), global fit index (GFI), comparative fit index (CFI), and normal fit index (NFI). The SEM showed a high goodness of fit when the indices matched the fitting conditions. Based on the standardized path coefficients obtained from model fitting, an intuitive understanding of the magnitude of each variable's effect was provided.

### 4.2 System dynamics (SD)

#### 4.2.1 Step 4: SD model establishment

SD models were created using the VENSIM PLE software to characterize the effects of SEM-based non-linearities. The model boundaries were first established by rationalizing the primary research variables included in the current research topic. The logical structure of the model was then developed, comprising a causal diagram and a stock-flow diagram, which interrelate the variables and express an understanding of the waste sorting behavioral process. Finally, equations expressing the interrelationships between the variables were established to lay the groundwork for predicting the potential system behavior over time (Vahidi and Aliahmadi, 2019).

#### 4.2.2 Step 5: SD model validation

After building the model, it must be verified to confirm that the simulation results of the SD model are consistent with the actual situation. The purpose of the SD model validation is to guarantee that the SD model is objectively justifiable and that the simulation results are referable. The SD model is frequently validated in three ways: structural consistency, dimensional consistency, and historical validity (Cao et al., 2024).

#### 4.2.3 Step 6: scenario simulation

After completing the model building and validation, simulations were used to create multiple scenarios by assigning different values to the variables to determine the dynamic process of influencing different factors on the change in waste sorting behavior.

## 5 Results

### 5.1 SEM results

#### 5.1.1 Demographic characteristics

Table 1 shows the demographic characteristics of the respondents. Of the participants, 46.4% were men and 53.6% were women. Based on the age distribution, young people aged 26–35 made up most of the total survey population, matching the demographic profile of Chinese Internet users. People with bachelor's degrees accounted for 75.1% of the total survey population, with those with monthly household earnings above 8,000 CNY making up the majority (52.2%).

**Table 1:** Demographic characteristics.

**Characteristics**	**Category**	**Frequency**	**Percentage (%)**
Gender	Male	257	46.4
	Female	297	53.6
Age	18–25 years	76	13.7
	26–35 years	277	50.0
	36–45 years	156	28.2
	46–55 years	37	6.7
	Abov 55 years	8	1.4
Educational level	High school below	8	1.4
	High school	22	4.0
	College degree	62	11.2
	Bachelor's degree	416	75.1
	Master's degree or above	46	8.3
Income per month^*^ (CNY)	Below 1,000	6	1.1
	1,001–3,000	43	7.8
	3,001–5,000	45	8.1
	5,001–8,000	171	30.9
	Above 8,000	289	52.2

CNY stands for Chinese Yuan.

#### 5.1.2 Reliability and validity tests

Table 2 shows the standardized Cronbach's α, CR, and AVE for each variable, as well as the factor loadings for its indicators. The questionnaire data demonstrated strong reliability, with a total Cronbach's alpha of 0.92, and each variable having a value greater than 0.7. The factor loadings of each measured indicator for variables ranged from 0.5 to 0.95, and each variable's composite reliability (CR) exceeded 0.7. The average variance extracted (AVE) value exceeded 0.5. The findings show that the convergent validity of the variables fits the research standards.

**Table 2:** Reliability and validity tests.

**Latent variables**	**Observed variables**	**Factor loading**	**Cronbach's α**	**AVE**	**CR**
KI	KI1	0.824	0.798	0.577	0.803
	KI2	0.668			
	KI3	0.779			
IF	IF1	0.789	0.867	0.621	0.867
	IF2	0.801			
	IF3	0.787			
	IF4	0.774			
SN	SN1	0.760	0.823	0.610	0.824
	SN2	0.799			
	SN3	0.783			
HA	HA1	0.776	0.827	0.614	0.827
	HA2	0.771			
	HA3	0.803			
IN	IN1	0.780	0.880	0.595	0.880
	IN2	0.793			
	IN3	0.783			
	IN4	0.771			
	IN5	0.729			
WSB	WSB1	0.740	0.871	0.532	0.872
	WSB2	0.759			
	WSB3	0.754			
	WSB4	0.763			
	WSB5	0.668			
	WSB6	0.687			

The square roots of the AVE values for all variables were greater than the correlation coefficients between them, showing strong discriminant validity. Table 3 shows the specifics of discriminant validity.

**Table 3:** Discriminant validity test.

**Latent variables**	**KI**	**IF**	**SN**	**HA**	**IN**	**WSB**
KI	**0.760**					
IF	0.230	**0.788**				
SN	0.295	0.579	**0.781**			
HA	0.289	0.621	0.606	**0.784**		
IN	0.193	0.414	0.427	0.476	**0.771**	
WSB	0.419	0.586	0.646	0.619	0.498	**0.729**

The value on the diagonal (bold value) represents the square root of each latent variable's AVE value.

#### 5.1.3 Goodness of fit and parameter estimation

**Figure 3:**
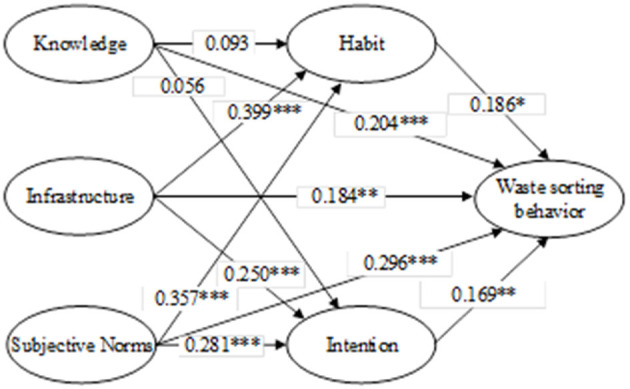
Results for the SEM model. ^*^Represents *p* < 0.05, ^**^represents *p* < 0.01, and ^***^represents *p* < 0.001.

Table 4 displays the primary fitness test indicators for model fit, demonstrating that the data and the SEM model were able to establish a good fit. Figure 3 further illustrates the validated path relationships. The analysis in Table 5 reveals that all the SEM model path hypotheses (H1-H11) are significantly established, except for the standard path coefficients of H6 and H7, which do not meet the p < 0.05 requirement and hence, they are not supported. It can be found that KI has a direct association with WSB but no indirect effect. The *R*^2^ of WSB was 0.601, and the factors in the model explained 60.1% of the WSB variation.

**Table 4:** Main fitness test indicators.

**Evaluation index**	**CMIN/DF**	**RMSEA**	**GFI**	**CFI**	**NFI**
Evaluation standard	< 3.000	< 0.080	>0.900	>0.900	>0.900
Model fit	1.689	0.035	0.944	0.975	0.940

**Table 5:** Standardized path coefficients.

**Hypothesis**	**Path**	**Estimate**	**S.E**.	**Hypothesis**
H1	KI → WSB	0.204	0.050	Accept
H2	IF → WSB	0.184	0.069	Accept
H3	SN → WSB	0.296	0.085	Accept
H4	HA → WSB	0.186	0.080	Accept
H5	IN → WSB	0.169	0.054	Accept
H6	KI → HA	0.093	0.055	Reject
H7	KI → IN	0.056	0.060	Reject
H8	IF → HA	0.399	0.077	Accept
H9	IF → IN	0.250	0.074	Accept
H10	SN → HA	0.357	0.079	Accept
H11	SN → IN	0.281	0.072	Accept

### 5.2 SD results

#### 5.2.1 Model development

**Figure 4:**
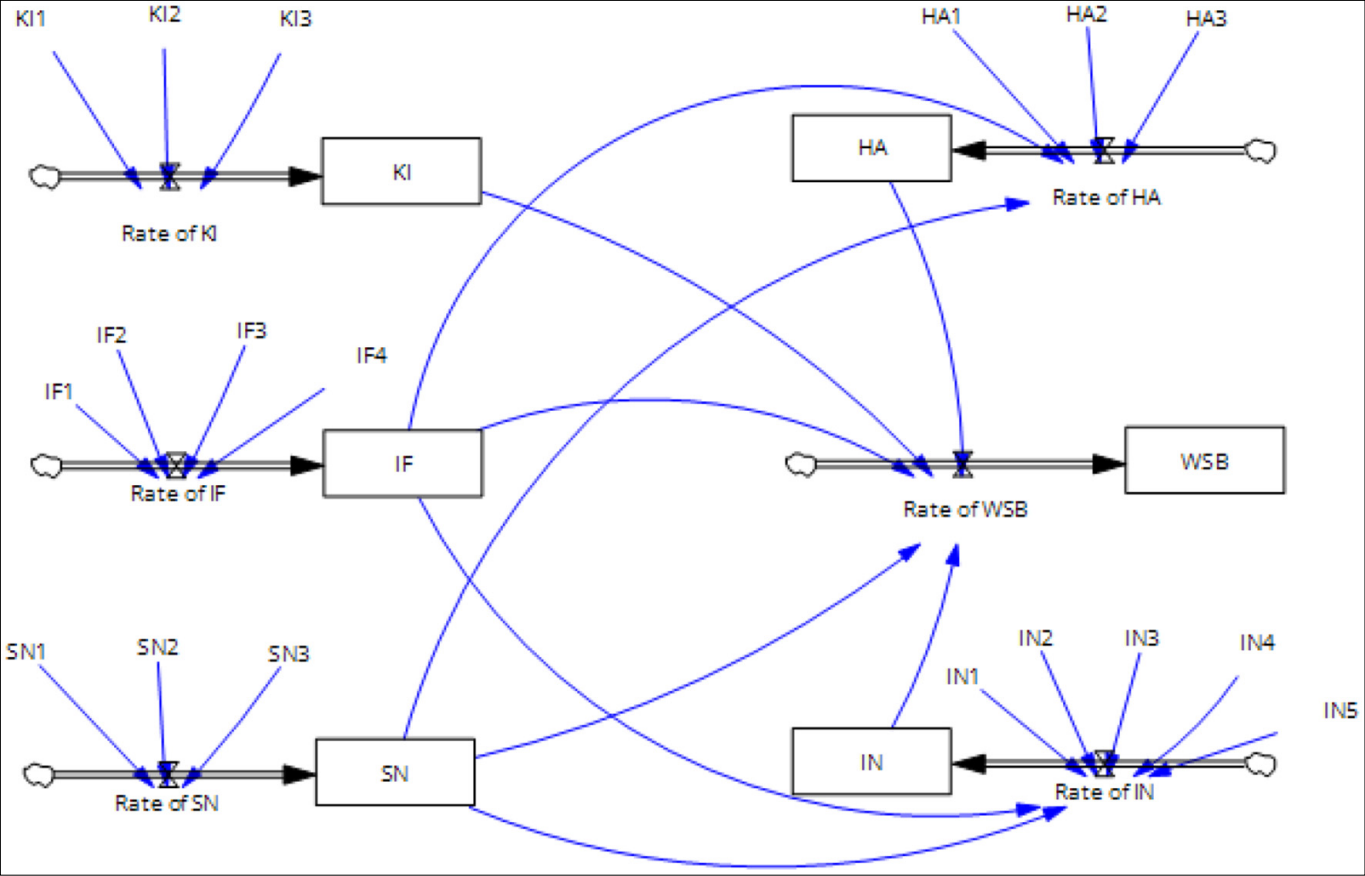
Stock flow diagram.

The SEM model identified five factors associated with residents' waste sorting behavior. Combined with the variables measured in the SEM model, six state variables, six rate variables, and 18 constants are identified in the SD model. The stock flow diagram (Figure 4) embodies the relationships between the variables and serves as the foundation for developing a relationship equation.

To create these equations, parameters derived from the SEM model were used to determine the values of the variables in the SD model. Standard path coefficients can be used to calculate the coefficients between state variables. The normalized weights of the constants were calculated, as listed in Table 6, to determine the effect coefficients between the constants and the rate variables. For example, in the SD model, the normalized weight of KI1 was 0.381, which was calculated as 0.870/(0.870 + 0.659 + 0.752) = 0.381. The initial values of the constants were the same as the *R*^2^ values in the SEM. The initial values of the state variables were the arithmetic means of the initial values of the constants under the same dimension. All equations are presented in Appendix B.

**Table 6:** SD model parameters.

**Variables**	**Standard regression coefficient in SEM**	**Weight**	***R* ^2^**	**Initial value**
KI				0.585
KI1	0.870	0.381	0.757	0.757
KI2	0.659	0.289	0.434	0.434
KI3	0.752	0.330	0.565	0.565
IF				0.660
IF1	0.809	0.249	0.654	0.654
IF2	0.813	0.250	0.661	0.661
IF3	0.834	0.257	0.696	0.696
IF4	0.792	0.244	0.627	0.627
SN				0.591
SN1	0.723	0.314	0.523	0.523
SN2	0.802	0.348	0.643	0.643
SN3	0.778	0.338	0.606	0.606
HA				0.648
HA1	0.804	0.333	0.647	0.647
HA2	0.789	0.327	0.623	0.623
HA3	0.820	0.340	0.673	0.673
IN				0.598
IN1	0.797	0.206	0.635	0.635
IN2	0.765	0.198	0.585	0.585
IN3	0.788	0.204	0.621	0.621
IN4	0.765	0.198	0.585	0.585
IN5	0.751	0.194	0.564	0.564
WSB				0.532

#### 5.2.2 Model stability test

A structural validity test ensured that the developed model was rational. The causal relationship between waste sorting behavior and its associated factors in the SD model was determined using SEM, proving the model's structural soundness.

A dimensional consistency test was run to confirm that each equation and parameter in the model had consistent dimensionality. The VENSIM PLE software includes an automatic detection feature for equation writing; if there is an issue with the equation input, an error message will show. During model development and simulation process, no error warning messages came in VENSIM, indicating that the model's dimensional consistency had been confirmed.

**Figure 5:**
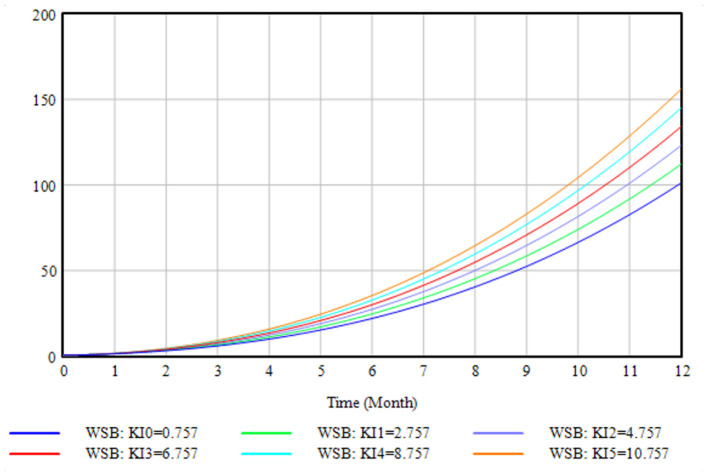
Historical validity test.

Historical validity testing determines how well the model simulation results match the actual system data. KI1 (knowledge of the importance of sorting) was randomly chosen as an example in the model, and its values were set to 0.757 (initial value), 2.757, 4.757, 6.757, 8.757, and 10.757 to demonstrate how waste sorting behavior was altered by KI1. As shown in Figure 5, the composite curves for the six values followed the same developmental trend, demonstrating that residents who were more conscious of the importance of waste sorting were more likely to practice waste sorting activities. This finding is consistent with the results of a previous theoretical investigation (Li et al., 2017), demonstrating that the established SD model appropriately reflects the effects of the variable changes.

#### 5.2.3 Simulation analysis

The SD model was configured as follows: initial time = 0, final time = 12, time step = 0.25, and time unit, months. The set model parameters were substituted into the related mathematical equations to track the dynamic changes and development of variables in the SD model.

**Figure 6:**
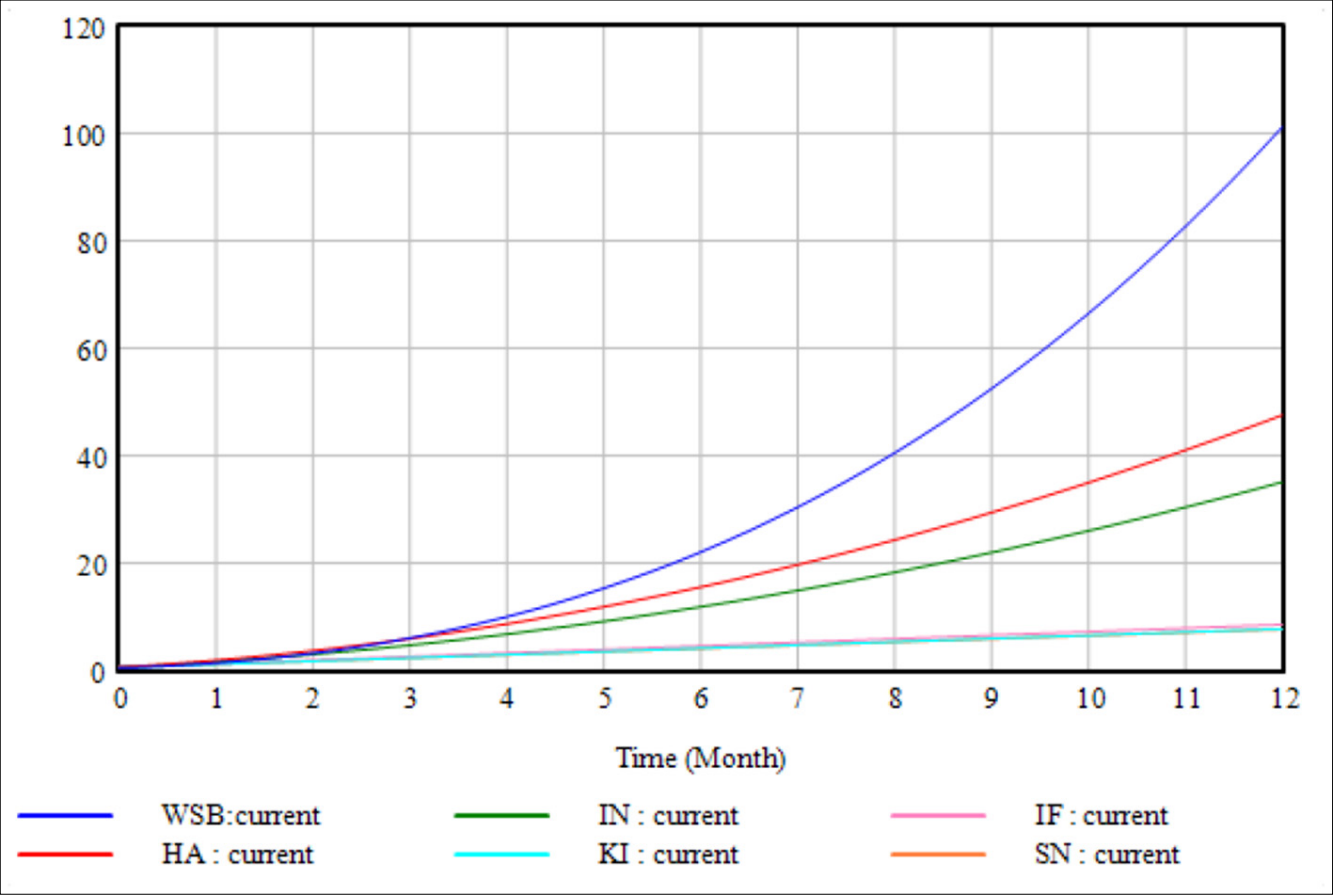
Base case scenario simulation.

Figure 6 depicts the trends in KI, IF, SN, HA, IN, and WSB during the simulation period. The base simulation results demonstrate that as time passes, KI, IF, SN, HA, and IN show a roughly linear growth tendency, with the ranges of changes in KI and SN being relatively close. The WSB grew curvilinearly with time and slowly in the first half of the simulation cycle before accelerating in the second half.

**Figure 7:**
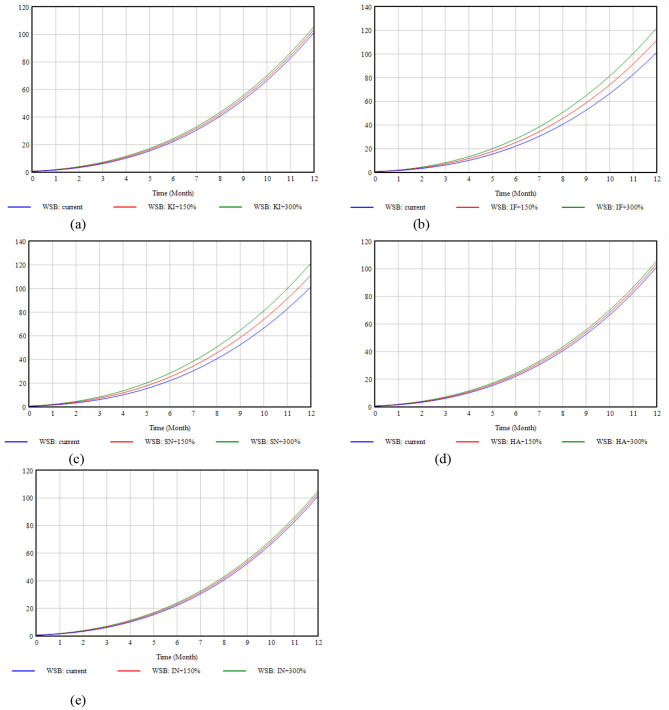
Scenario simulation. **(a)** Impact of KI changes on WSB. **(b)** Impact of IF changes on WSB. **(c)** Impact of SN changes on WSB. **(d)** Impact of HA on WSB. **(e)** Impact of IN changes on WSB. KI + 300% → WSB = 105.532, IF + 300% → WSB = 121.865, SN + 300% → WSB = 121.195, HA + 300% → WSB = 105.575, IN + 300% → WSB = 104.874.

To further explore the effects of KI, IF, SN, HA, and IN on WSB, the initial values of these variables were adjusted to observe their effects on the simulation results. Five scenarios were generated by increasing the initial value of a single variable by 150% and 300% while keeping the other variables constant. Figure 7 shows the simulation results for this scenario. The findings revealed that WSB increased significantly as the KI, IF, SN, HA, and IN levels increased. According to the WSB values obtained after a 300% increase in each element, the decreasing order of factor influence utility was IF > SN >HA > KI > IN.

## 6 Discussion

Unlike previous studies that qualitatively identify the associated factors of waste sorting behavior based on the COM-B framework (Allison et al., 2021; Cook et al., 2023), this study explores the static and dynamic relationships between waste sorting behavior and its associated factors. Using the SEM method, the effect strengths among the components of the COM-B model were accurately quantified. Based on these results combined with the SD model, a system mapping association of waste sorting behavior was established. This integrated approach reduces the errors and biases from expert judgment in systematic modeling, and reveals the dynamic evolutionary trend of behavior-associated factors, transforming COM-B model from a descriptive classification framework into a predictive tool for forecasting the evolution of waste sorting behavior.

By testing 11 theoretical hypotheses, it was discovered that the components of capability, opportunity, and motivation, demonstrate direct associations with WSB. The results concur with those of Ng et al. (2021) and Zhang et al. (2021), who found that increasing KI, IF, SN, HA, and IN directly encouraged residents to implement more waste sorting. Meanwhile, IF and SN have indirectly associations with WSB via HA and IN, as verified by Li et al. (2017), Liang et al. (2024), and Liu et al. (2024). Thus, raising IF and SN can aid in the creation of HA while also inspiring IN by assessing the cost of sorting and finally improving WSB.

The study reveals while knowledge demonstrates a significant direct association with waste sorting behavior, it has no mediating linkage to behavior through habit or intention (H6/H7 was rejected). This is incongruent with the COM-B model's theoretical assumptions, which can be interpreted in two ways. First, habit formation initially needs to be goal-driven to sustain behavioral repetition (Wood and Rünger, 2016). Educational and promotional activities in Shanghai communities have focused mainly on the dissemination of operational knowledge (e.g., standards and steps for waste sorting), while neglecting value-oriented knowledge output (e.g., the environmental value of waste sorting). Thus, residents have acquired operational skills but lack internalized environmental values to form sustainable goals. Finally, ignoring the “value-shaping” properties of knowledge may inhibit habit formation. Concurrently, waste sorting in China is a policy-mandated activity rather than a fully voluntary environmental initiative. Numerous supporting policies have been implemented in Shanghai, including community supervision and fine systems. When policy constraints are sufficiently strong, residents may directly interpret the knowledge they possess as action instructions without undergoing a strong intention-forming process. For example, Shanghai residents who know that “dry waste goes in the black bin” act on that knowledge without much deliberative processing. Therefore, external policy constraints allow knowledge to circumvent intention and have a direct effect on behavior.

Opportunity factors demonstrate the strongest statistical association with WSB, followed by capability and motivation. These findings support Meng (2019), who concluded that the combined effect of external opportunity variables outweighs the capability and motivation components resulting from individual subjectivity. Such as the broad availability of well-designed, properly labeled, and simple-to-use recycling containers, considerably lowers the cost of implementing recycling practices, making them practical. Similarly, the proactive practice of waste sorting by key reference groups (such as neighbors, community leaders, and public figures), combined with stated expectations and obligations for waste sorting within the community or society, generates intangible social pressure (Sarkis, 2017). The press and obligation motivate residents to change their conduct to meet group expectations, resulting in active participation in waste sorting. Therefore, policy design and behavioral interventions should concentrate on systematically creating supportive opportunity environments.

During the simulation period, the non-linear growth trend of waste sorting behavior (WSB) from slow to fast indicates that the behavior diffusion process may be influenced by the cumulative impacts of external stimuli. Specifically, the levels of the five basic factors—KI, IF, SN, HA, and IN—rise over time, and their marginal impacts on WSB show a growing tendency. For example, once infrastructure coverage reaches a particular level or social norms begin to emerge, further improvements in subsequent factors may more easily trigger significant behavioral leaps. As a result, when developing and implementing strategies to encourage waste sorting behavior, it is critical to consider the timing of intervention as well as the current state of the system, acknowledging that early investments may lay the groundwork for more significant behavioral changes later on, thereby optimizing resource allocation and strategy design.

### 6.1 Managerial implications

Given that opportunity factors demonstrate the statistically strongest association with behavior, strategies to improve them can produce higher behavioral facilitation results than capability and motivation components. The Behavior Change Wheel Theory provides a solid foundation for designing interventions in addressing the opportunity component. These interventions include reducing obstacles to using waste sorting facilities by expanding facility coverage, optimizing facility appearance design, improving ease of facility operation, and flexibly setting centralized drop-off times. Second, it minimizes residents' options for non-segregation from both hardware and software perspectives, such as prohibiting traditional waste cans as soon as it is feasible and organizing patrols to stop and advise residents on non-segregation behavior. Third, local government can select benchmark communities, model families, and individuals for waste sorting; provide publicity, commendation, and material rewards to the winners; maximize the effect of exemplary role models; and motivate other residents to participate in waste sorting.

### 6.2 Limitation and future research

This study has some limitations. It investigates only a part of the capability, opportunity, and motivational elements relevant to waste sorting among urban residents. Future research could thus expand the framework by incorporating additional components, such as personal energy under capacity and economic or ecological benefits under motivation factors. In addition, the study focuses on Shanghai, which is among the top cities in the country in terms of the degree of refinement and implementation of waste sorting policies. However, policy variability across cities can differentiate the mechanisms of residents' waste sorting behavior (Tian et al., 2022). The generalizability of the findings to cities with imperfect policies is questionable. Therefore, it is proposed that future studies select diverse city samples to assess the model's applicability across varying policy contexts and develop precisely behavior promotion programs. Finally, although a strict sampling method was used to collect the data, the limitations of the web-based survey resulted in a younger and more highly educated sample. Therefore, field sampling data can be supplemented in future work to improve sample representativeness.

## 7 Conclusion

The efficient promotion of urban waste sorting in China is inextricably linked to the active participation of residents in source sorting. Based on the COM-B theory, this study used a combination of SEM and SD methodologies to identify the key factors associated with residents' waste sorting behaviors and to depict the dynamic evolution of the behavior. The conclusions of this study are as follows.

First, the model shows that capability, opportunity, and motivation factors are directly related to waste sorting behavior, and opportunity also has an indirect effect on behavior via motivation. Second, specific emphasis should be placed on IF and SN, which are opportunity factors, and prioritizing interventions to improve them can help encourage more successful waste sorting. Third, post-management is crucial, as the quantitative change of these factors will result in a qualitative change of behavior during this stage. This study extends the boundaries of theoretical applications and enriches the research perspective, while providing valuable insights and references for promoting the implementation of waste sorting among urban residents.

## Data Availability

The raw data supporting the conclusions of this article will be made available by the authors, without undue reservation.
