# Supplementary material for: What drives waste sorting? A capability, opportunity, motivation, and behavior model analysis with hybrid modeling
Source: Front Psychol. 2025 Sep 15;16:1625538. doi: 10.3389/fpsyg.2025.1625538 (PMC12477212; doi:10.3389/fpsyg.2025.1625538)
Supplement: Supplementary file 1 [file Table_1.docx]

**Appendix**

**A: Questionnaire items**

| Variable | Item | Source |
| --- | --- | --- |
| KI | I know the importance of waste sorting for environmental protection.  I know the operation process of waste sorting.  I know how to categorize waste correctly. | Zhang et al  ([2021](file:///D:\安徽大学教师科研工作\已发表及待发论文\垃圾分类待完成论文\What%20Drives%20Waste%20Sorting%20A%20COM-B%20Model%20Analysis%20with%20Hybrid%20Modeling\What%20Drives%20Waste%20Sorting%20A%20COM-B%20Model%20Analysis%20with%20Hybrid%20Modeling\Frontiers%20in%20Psychology\Manuscript%20with%20clean%20version.docx#Zhang)) |
| IF | I think the number of waste sorting collection facilities in the district is sufficient.  I think the waste sorting signs on the waste sorting collection facilities in my neighborhood are clearly printed.  I think it is easy to get to the waste sorting collection points in my neighborhood.  I think the waste sorting facilities in my neighborhood are relatively clean. | Xu et al ([2017](file:///D:\安徽大学教师科研工作\已发表及待发论文\垃圾分类待完成论文\What%20Drives%20Waste%20Sorting%20A%20COM-B%20Model%20Analysis%20with%20Hybrid%20Modeling\What%20Drives%20Waste%20Sorting%20A%20COM-B%20Model%20Analysis%20with%20Hybrid%20Modeling\Frontiers%20in%20Psychology\Manuscript%20with%20clean%20version.docx#Xu2017)) |
| SN | My family members categorize their waste.  My friends categorize their waste.  All my neighbors in my neighborhood categorize their waste. | Ajzen ([1991](file:///D:\安徽大学教师科研工作\已发表及待发论文\垃圾分类待完成论文\What%20Drives%20Waste%20Sorting%20A%20COM-B%20Model%20Analysis%20with%20Hybrid%20Modeling\What%20Drives%20Waste%20Sorting%20A%20COM-B%20Model%20Analysis%20with%20Hybrid%20Modeling\Frontiers%20in%20Psychology\Manuscript%20with%20clean%20version.docx#Ajzen)) |
| HA | I am used to doing waste sorting every day  I insist on doing waste sorting every day  I consciously do waste sorting every day. | Li et al ([2017](file:///D:\安徽大学教师科研工作\已发表及待发论文\垃圾分类待完成论文\What%20Drives%20Waste%20Sorting%20A%20COM-B%20Model%20Analysis%20with%20Hybrid%20Modeling\What%20Drives%20Waste%20Sorting%20A%20COM-B%20Model%20Analysis%20with%20Hybrid%20Modeling\Frontiers%20in%20Psychology\Manuscript%20with%20clean%20version.docx#Li)) |
| IN | I am willing to categorize waste in the future.  I have plans to categorize waste in the future.  If possible, I will advise others to categorize their waste.  If necessary, I will help the older generation (parents/parents-in-law) in my family to categorize waste.  If necessary, I will teach my next generation (children) to categorize waste. | Ajzen ([1991](file:///D:\安徽大学教师科研工作\已发表及待发论文\垃圾分类待完成论文\What%20Drives%20Waste%20Sorting%20A%20COM-B%20Model%20Analysis%20with%20Hybrid%20Modeling\What%20Drives%20Waste%20Sorting%20A%20COM-B%20Model%20Analysis%20with%20Hybrid%20Modeling\Frontiers%20in%20Psychology\Manuscript%20with%20clean%20version.docx#Ajzen))  Lou et al ([2024](file:///D:\安徽大学教师科研工作\已发表及待发论文\垃圾分类待完成论文\What%20Drives%20Waste%20Sorting%20A%20COM-B%20Model%20Analysis%20with%20Hybrid%20Modeling\What%20Drives%20Waste%20Sorting%20A%20COM-B%20Model%20Analysis%20with%20Hybrid%20Modeling\Frontiers%20in%20Psychology\Manuscript%20with%20clean%20version.docx#Lou)) |
| WSB | In the past year, I ______ categorized roots and leaves and peels.  In the past year, I_______ categorized leftovers.  In the past year, I ______ categorized waste paper products.  In the past year, I ______ categorized plastic products.  In the past year, I ______ categorized glass products.  In the past year, I ______ categorized used batteries. | Hopper &  Nielsen ([1991](file:///D:\安徽大学教师科研工作\已发表及待发论文\垃圾分类待完成论文\What%20Drives%20Waste%20Sorting%20A%20COM-B%20Model%20Analysis%20with%20Hybrid%20Modeling\What%20Drives%20Waste%20Sorting%20A%20COM-B%20Model%20Analysis%20with%20Hybrid%20Modeling\Frontiers%20in%20Psychology\Manuscript%20with%20clean%20version.docx#Hopper)) |

**B: Equations of the SD model**

- *Rate variables*

(1) Rate of KI=KI1*0.381+KI2*0.289+KI3*0.330

(2) Rate of IF=IF1*0.249+IF2*250+IF3*0.257+IF4*0.244

(3) Rate of SN=SN1*0.314 + SN2*0.348+SN3*0.338

(4) Rate of HA=HA1*0.333+HA2 *0.327+HA3*0.34+IF*0.399+SN*0.357

(5) Rate of IN=IN1*0.206+IN2*0.198+IN3*0.204+IN4*0.198+IN5*0.194+ IF *0.25+SN*0.281

(6) Rate of WSB=HA*0.186+IF*0.184+IN*0.169+KI*0.204+SN*0.269

- *State variables*

(7) KI=INTEG (Rate of KI)

(8) IF=INTEG (Rate of IF)

(9) SN=INTEG (Rate of SN)

(10) HA=INTEG (Rate of HA)

(11) IN=INTEG (Rate of IN)

(12) WSB=INTEG (Rate of WSB)
